# OprF functions as a latch to direct outer membrane vesicle release in *Pseudomonas aeruginosa*

**DOI:** 10.1128/mbio.01017-26

**Published:** 2026-07-16

**Authors:** Shrestha Mathur, Citrupa Gopal, Susan K. Erickson, Leah R. Goldberg, Sonia Hills, Abigail G. B. Radin, Jeffrey W. Schertzer

**Affiliations:** 1 Department of Biological Sciences, Binghamton University 14787, Binghamton, New York, USA; 2 Department of Cell Biology and Molecular Genetics, Maryland Pathogen Research Institute, University of Maryland 1068 , College Park, Maryland, USA; 3 Binghamton Biofilm Research Center, Binghamton University 14787, Binghamton, New York, USA; Carnegie Mellon University, Pittsburgh, Pennsylvania, USA

**Keywords:** outer membrane vesicles, OprF, OmpA family, *Pseudomonas aeruginosa*, outer membrane proteins, porin, protein conformation

## Abstract

Bacterial outer membrane vesicles (OMVs) contribute to virulence, competition, immune avoidance, and communication. This has led to great interest in how they are formed. To date, investigation has focused almost exclusively on what controls the initiation of OMV biogenesis. Regardless of the mechanism of initiation, all species face a similar challenge before an OMV can be released: how does the outer membrane (OM) detach from the underlying peptidoglycan (PG) in regions that will ultimately bulge and then vesiculate? The OmpA family of OM proteins (OprF in *Pseudomonas aeruginosa*) is widely conserved and unusually abundant in OMVs across species, considering their major role in PG attachment. OmpA homologs also have the interesting ability to adopt both PG-bound (two-domain) and PG-released (one-domain) conformations. Using targeted deletion of the PG-binding domain, we show here that loss of cell wall association, not general membrane destabilization, is responsible for hypervesiculation in OprF-modified strains. We therefore propose that OprF functions as a “latch,” capable of releasing PG in regions destined to become OMVs. To test this hypothesis, we developed a protocol to assess OprF conformation in live cells and purified OMVs. While >90% of OprF proteins exist in the two-domain conformation in the OM of cells, we show that the majority of OprF in OMVs is present in the one-domain conformation. With this work, we take some of the first steps in characterizing late-stage OMV biogenesis and identify a family of proteins whose critical role can be explained by their unique ability to fold into two distinct conformations.

**IMPORTANCE:**

Vesicular transport is now recognized to operate in all domains of life. Nevertheless, the study of outer membrane vesicle (OMV) biogenesis has been challenging because genetic screens failed to identify proteins analogous to those involved in eukaryotic vesicular transport. With this work, we identify the first protein whose direct action can both define the location and govern the mechanism of OMV release. Our latch model is consistent with previous observations linking OmpA family proteins to OMV biogenesis but further describes a physical mechanism that has broad implications for vesicle production and function across species. The work presented here unifies disparate OMV biogenesis mechanisms at the point of vesicle release and underscores the stark differences in how transport vesicles are formed in prokaryotes vs eukaryotes.

## INTRODUCTION

Bacterial production of outer membrane vesicles (OMVs) is now recognized to be ubiquitous. OMVs range in size from 50 to 300 nm in diameter, are comprised of outer membrane (OM) components released from the parent cell, and transport diverse and seemingly regulated cargo (1). In fact, vesicular transport has been recognized as a distinct bacterial secretion system (2). The ability of OMVs to transport cargo at high concentration over long distances while protected from the environment makes them well suited for many functions. These include virulence (3, 4), interbacterial competition (5), immune modulation/avoidance (6, 7), horizontal gene transfer (8), cell-cell communication (9), nutrient acquisition (10, 11), and biofilm development (12, 13).

Several models have been proposed to describe OMV formation in different species (1, 14). These involve peptidoglycan (PG) homeostasis (15, 16), stress response (17, 18), manipulation of membrane lipid composition (19, 20), intercalation of membrane-active small molecules (21, 22), and outright cellular explosion (14). Previous work has focused largely on the initiation of OMV biogenesis, particularly on how the membrane is induced to bulge. For example, we developed the bilayer-couple model (23) to explain how accumulation of membrane intercalating agents in one leaflet of the membrane relative to the other will cause the membrane to curve. Our model originally described the action of the OMV inducer Pseudomonas Quinolone Signal (PQS) produced by *Pseudomonas aeruginosa* (23), but the principle has also been applied to other systems (20). With the current work, we set out to explore later stages in OMV development, specifically how regions of the OM intended for release as OMVs detach from the underlying PG layer. OMV production has loosely been associated with the idea of loss of OM-PG linkage for some time (24–26). However, the nature of investigation in this area has often made it difficult to distinguish between effects on natural OMV biogenesis pathways and unrelated membrane shedding or cellular disintegration.

OM-PG anchoring proteins tend to be excluded from the cargo of OMVs (27). Interestingly, this is not true for the widely conserved OmpA family. Although these proteins have a well-established role in OM-PG linkage (28, 29), they are abundantly packaged into the OMVs of several species (30, 31). This discrepancy might be explained by the unique ability of these proteins to adopt two distinct conformations. OmpA and its homolog OprF in *P. aeruginosa* are examples of “slow porins” found in the OM of a wide variety of Gram-negative species. They can fold into a beta-barrel structure that produces a large-diameter pore, but display remarkably low permeability when reconstituted into liposomes or planar bilayers (32). These seemingly contradictory observations are reconciled by the fact that OmpA homologs naturally fold into two conformers: a “closed” two-domain conformation where a C-terminal globular domain extends into the periplasm to interact with PG (28, 29, 33, 34), and an “open” one-domain conformation where the C-terminal region joins the N-terminal region embedded in the membrane to produce the large beta-barrel pore. Studies in *Escherichia coli* and *P. aeruginosa* showed that >90% of OmpA and OprF found in the OM exist in the “closed” two-domain conformation (35, 36), explaining the major role of these proteins in OM-PG linkage, as well as the low permeability of solutes through these proteins in cells and reconstituted systems.

In this study, we test the hypothesis that OprF in *P. aeruginosa* facilitates OMV production through its ability to exist in two different conformations. First, we demonstrate that specific loss of PG-interaction ability explains hypervesiculation in *oprF* mutant strains, rather than general membrane instability caused by the loss of this abundant OM protein. We then develop a methodology to measure the conformational distribution of OprF in cellular membranes vs OMVs and use this technique to discover that the ratio of the one- to two-domain conformers is inverted in OMVs relative to cells, with the vast majority in OMVs adopting the one-domain structure. We propose that OprF functions as a “latch” to direct OMV release by reducing the local density of OM-PG linkages in regions of the OM destined to become OMVs.

## RESULTS

### OprF variants properly translocate to the OM and correctly fold

**Fig 1:**
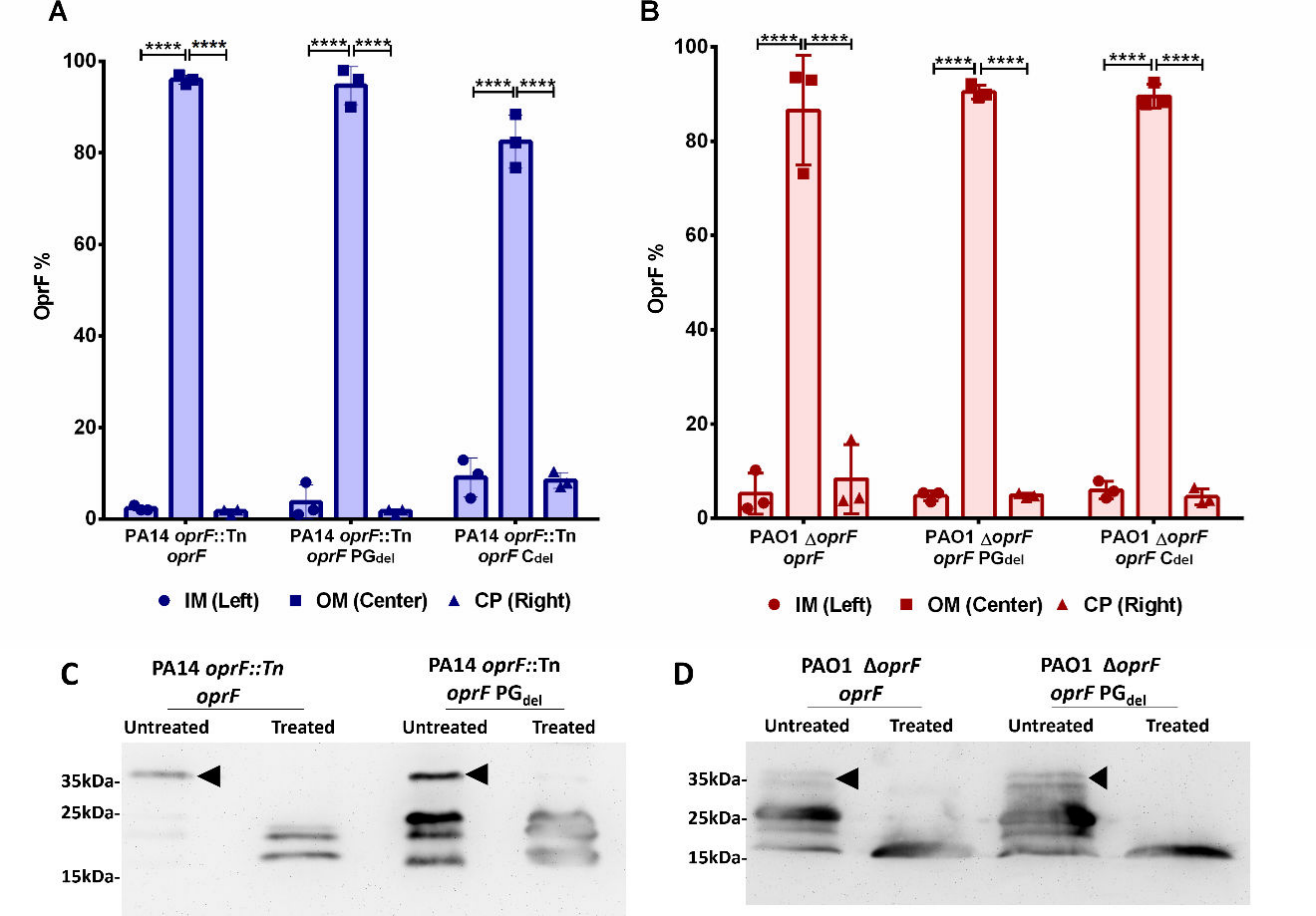
Validation of OprF variant translocation and conformation in the OM. The proportion of OprF variant proteins present in the IM, OM, and CP after expression in the PA14 (**A**) and PAO1 (**B**) *oprF* null strains was assessed by western blot (also see Fig. S2). Error bars represent standard deviation. Statistical significance was analyzed by two-way ANOVA, followed by Tukey’s multiple comparisons test (****P < 0.0001; *n* = 3). Purified OM fractions from the PA14 (**C**) and PAO1 (**D**) *oprF* null strains expressing full-length or PG_del_ OprF variants were subjected to proteolysis by proteinase K and analyzed by western blot using an N-terminal specific monoclonal antibody. The full-length OprF is marked by black arrows in the untreated lanes, while degraded OprF fragments are seen between 15 and 25 kDa after treatment with proteinase K. Blots are representative of three independent experiments.

We first created two variant OprF proteins: OprF ∆N_265_-V_276_ (OprF-PG_del_) is missing the majority of its PG-association motif (29, 37), while OprF ∆K_164_-K_326_ (OprF-C_del_) is missing the entire C-terminal domain (which contains the PG-association motif). Before testing whether either variant could rescue the *oprF*-null hypervesiculation phenotype (38), we needed to confirm that the variant proteins were properly translocated to the OM and correctly folded. Throughout this work, we conducted experiments in two common laboratory strains: PA14 and PAO1 (Table S1). PA14 was made *oprF*-null via transposon insertion into the *oprF* gene sequence, while PAO1 was made *oprF*-null through clean gene deletion. Western blots in Fig. 1A and B show that the vast majority of each OprF variant was found in the OM of both the PA14 and PAO1 mutant strains. See Fig. S1 for confirmation that our protocol effectively separates OM from inner membrane (IM) and cytoplasm (CP).

To assess proper folding of the OprF variant proteins, we employed a proteolysis protection assay performed on purified OM from *oprF* null mutant strains expressing each OprF variant. It is reported that 95% of OprF in the OM adopts the two-domain conformation in wild-type cells (36). Proteinase K treatment of purified OM fragments would therefore be expected to degrade the exposed C-terminal domain while leaving the OM-embedded N-terminal domain intact. Since OprF-C_del_ is missing the entire C-terminal domain and previous reports have confirmed that it folds correctly when expressed in *trans* (39), this analysis was only performed for native OprF and OprF-PG_del_. Figures 1C and D confirm that the native and PG_del_ OprF proteins were completely degraded from the ~35 kDa full-length protein to leave only the ~18 kDa N-terminal domain. Figure S2 shows that the OprF-C_del_ construct is of similar size, as expected. It is noteworthy that two common issues regarding OmpA family proteins are evident in the western blots presented. First, the doublet observed at ~35 kDa in Fig. 1D is characteristic of the “heat modifiability” of OprF and related proteins that arises from sometimes incomplete unfolding of the protein when heated in SDS (40–42). Second, the smaller bands present in the absence of proteinase K give evidence of the documented susceptibility of OprF to partial degradation near the domain boundary during cell lysis and handling (43). Nevertheless, our analysis confirms the work of Sugawara et al. (36), who first demonstrated that OprF is found almost exclusively in the two-domain conformation in the OM of cells.

### OprF variants fail to rescue the *oprF*-null hypervesiculation phenotype

**Fig 2:**
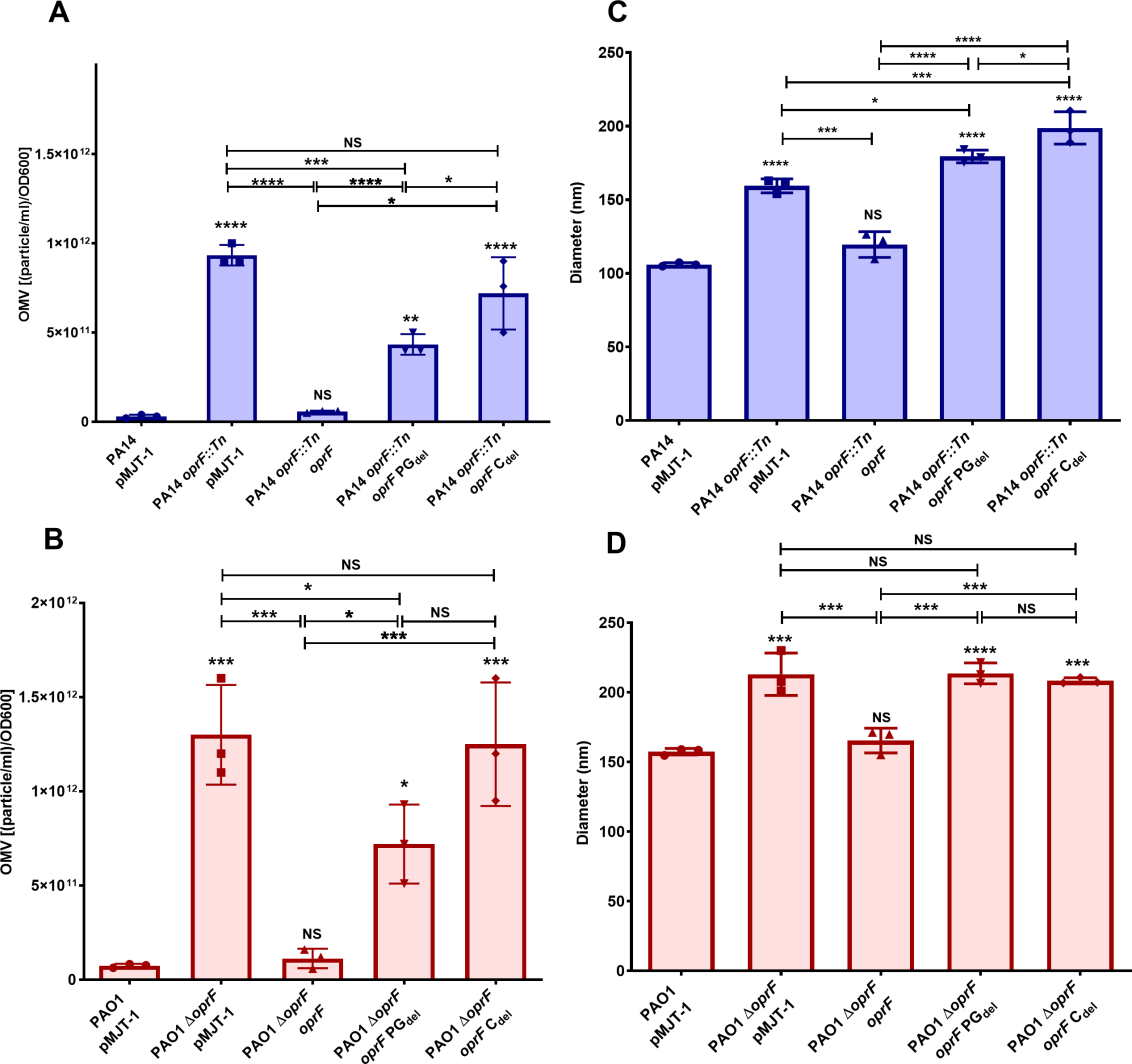
OMV quantity and size. OMVs were harvested from the identified strains and analyzed by nanoparticle tracking analysis (NTA). Vesicle quantity is presented in panels **A and B**. The mode of the vesicle size distribution is presented in panels **C and D**. Error bars represent the standard deviation. Statistical significance was analyzed by one-way ANOVA, followed by Tukey’s multiple comparisons test (*P < 0.05; **P < 0.01; ***P < 0.001; ****P < 0.0001; NS, not significant; *n* = 3). Asterisks directly over bars denote differences from WT; asterisks associated with horizontal lines refer to differences between the indicated bars.

Complete removal of OprF produces a known hypervesiculation phenotype (38). We used our targeted OprF sequence variants to address whether the specific loss of OprF-PG interaction was responsible for this hypervesiculation. Expression of native OprF in both the PA14 and PAO1 null mutant backgrounds resulted in complete rescue of the hypervesiculation phenotype (Fig. 2A and B). Both the OprF-PG_del_ and OprF-C_del_ variants failed to rescue the hypervesiculation phenotype, with OprF-C_del_ being indistinguishable from the *oprF* null mutant and OprF-PG_del_ showing partially reduced hypervesiculation.

Nanoparticle tracking analysis (NTA) of purified vesicles revealed that the mode diameter of OMVs produced by the *oprF* null mutants was significantly larger than that of the wild type. The pattern of rescue of this phenotype by expression of native or variant OprF proteins matched that for the hypervesiculation phenotype, suggesting that the ability of OprF to attach to PG is involved in controlling OMV size, as well as the level of OMV production (Fig. 2C and D).

### Hypervesiculation is not due to increased cell lysis

**Fig 3:**
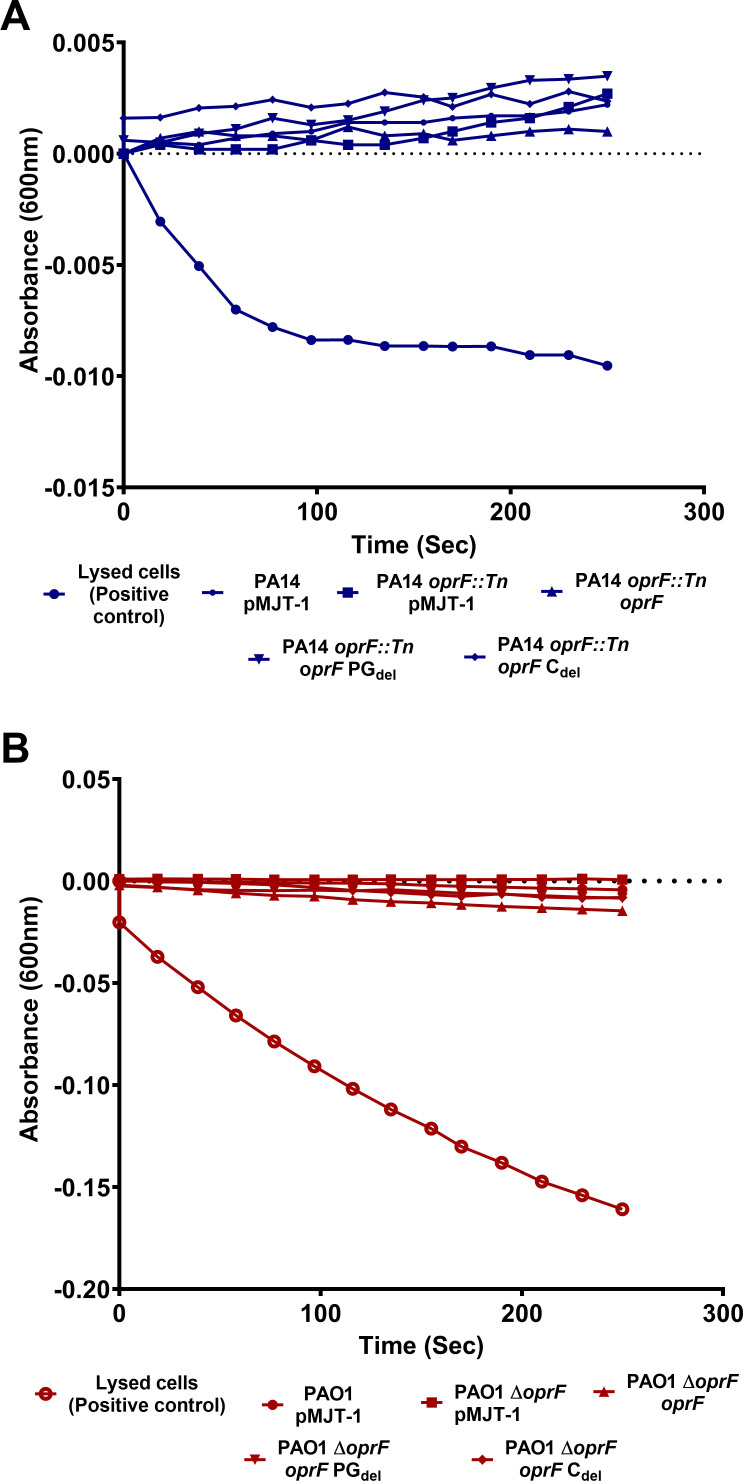
Succinate dehydrogenase activity. SDH enzyme activity was measured in OMVs prepared from all strains in both the PA14 (**A**) and PAO1 (**B**) backgrounds and compared to lysed bacterial cells (positive control). SDH assay results are representative of three independent experiments.

To ensure that the hypervesiculation phenotypes were not the result of increased cell lysis, we analyzed the purified OMVs for the presence of succinate dehydrogenase (SDH) enzyme activity. SDH is an integral inner membrane protein, and its presence in OMV preps would provide evidence of cell lysis. Figure 3 shows that no SDH activity was detected in the OMV preps from any of the PA14 or PAO1 background strains, demonstrating that hypervesiculation was not due to cellular disintegration or explosive lysis (14). This result is consistent with Wessel et al. (38), whose proteomic analysis showed that increased vesiculation in *oprF* mutants is not due to cell lysis. In further support, growth in liquid medium did not deviate from WT for any strain used in this study (Fig. S3). We went on to test every OMV prep analyzed in this study for SDH activity as a quality control step (Fig. S5 to S7).

### Hypervesiculation is not due to overproduction of PQS

**Fig 4:**
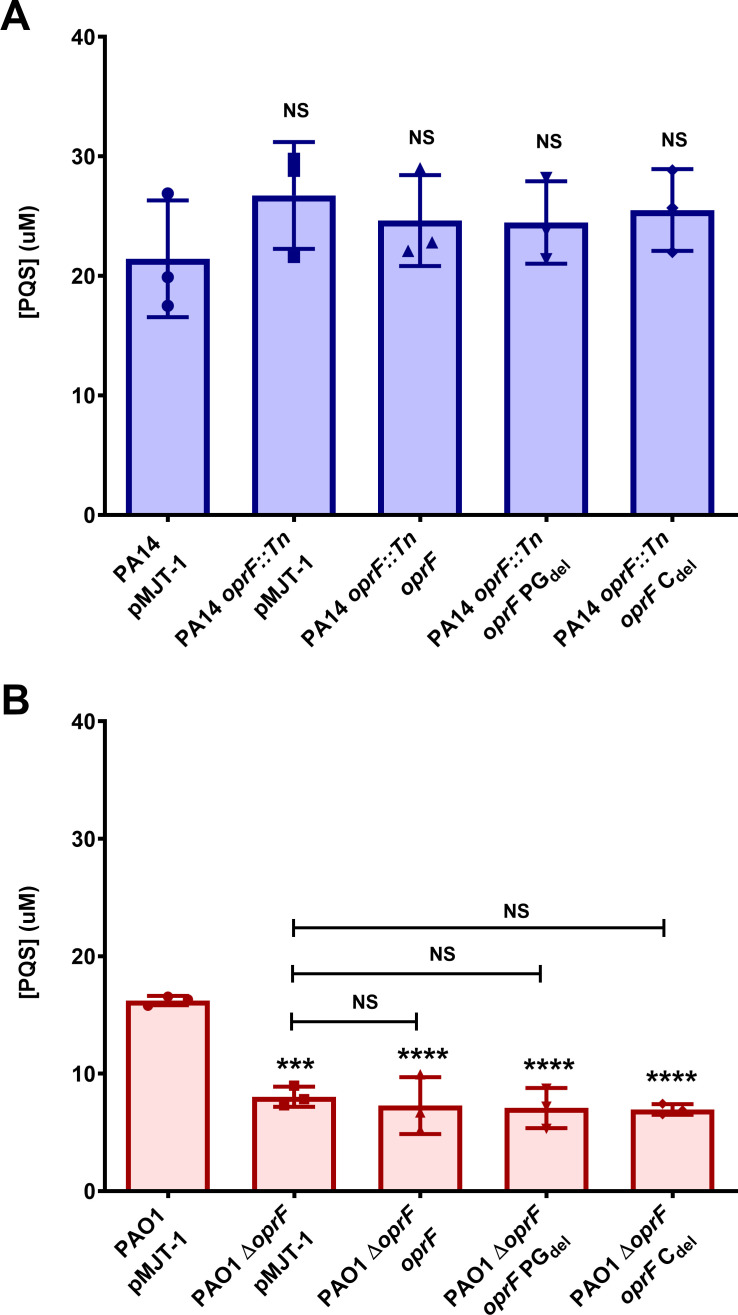
PQS production. PQS was extracted from all strains in both the PA14 (**A**) and PAO1 (**B**) backgrounds and quantified using thin-layer chromatography. Error bars represent standard deviation. Statistical significance was analyzed by one-way ANOVA, followed by Dunnett’s or Tukey’s multiple comparisons test (***P < 0.001; ****P < 0.0001; NS, not significant; *n* = 3). Asterisks directly over bars denote differences from WT; notations associated with horizontal lines refer to differences between the indicated bars.

Complete loss of OprF was previously shown to result in hypervesiculation, but attribution of mechanism was confounded by an accompanying increase in PQS production in the mutant strain (38). This was an issue because PQS is a known inducer of OMV formation (44–47). Here, we directly measured PQS production in each of our strains, but saw no increase. PQS production in the PA14 mutant strains was indistinguishable from WT (Fig. 4A). In PAO1, all mutant strains unexpectedly produced less PQS than WT, though still within the typical physiological range (Fig. 4B). Thus, hypervesiculation was not due to overproduction of PQS in our system.

### OprF is equally sorted between the OM and OMVs

**Fig 5:**
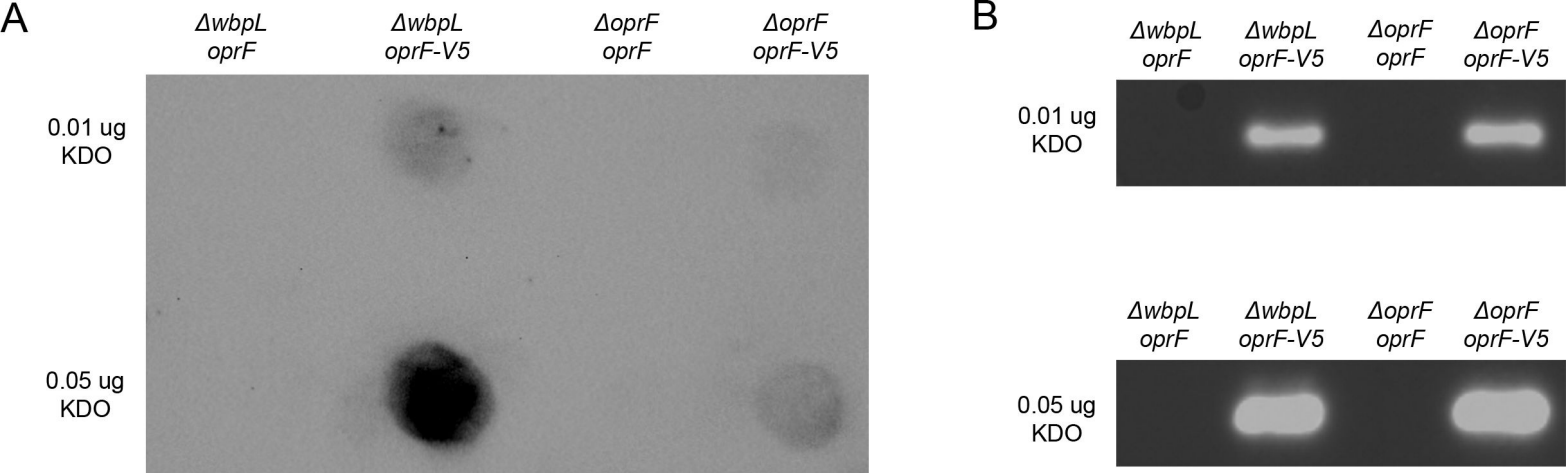
Surface accessibility of anti-OprF-V5 antibody. (**A**) Representative dot-blot comparing accessibility of the anti-V5 antibody to surface-exposed OprF-V5 on O-antigen negative (PAO1 ∆*wbpL* pMJT1-*oprF-V5*) and O-antigen positive (∆*oprF* pMJT1-*oprF-V5*) intact OMVs. PAO1 ∆*wbpL* pMJT1-*oprF* and ∆*oprF* pMJT1-*oprF* are negative controls. (**B**) Representative western blot confirming that an equal amount of total OprF-V5 protein is present in the OMV samples.

**Fig 6:**
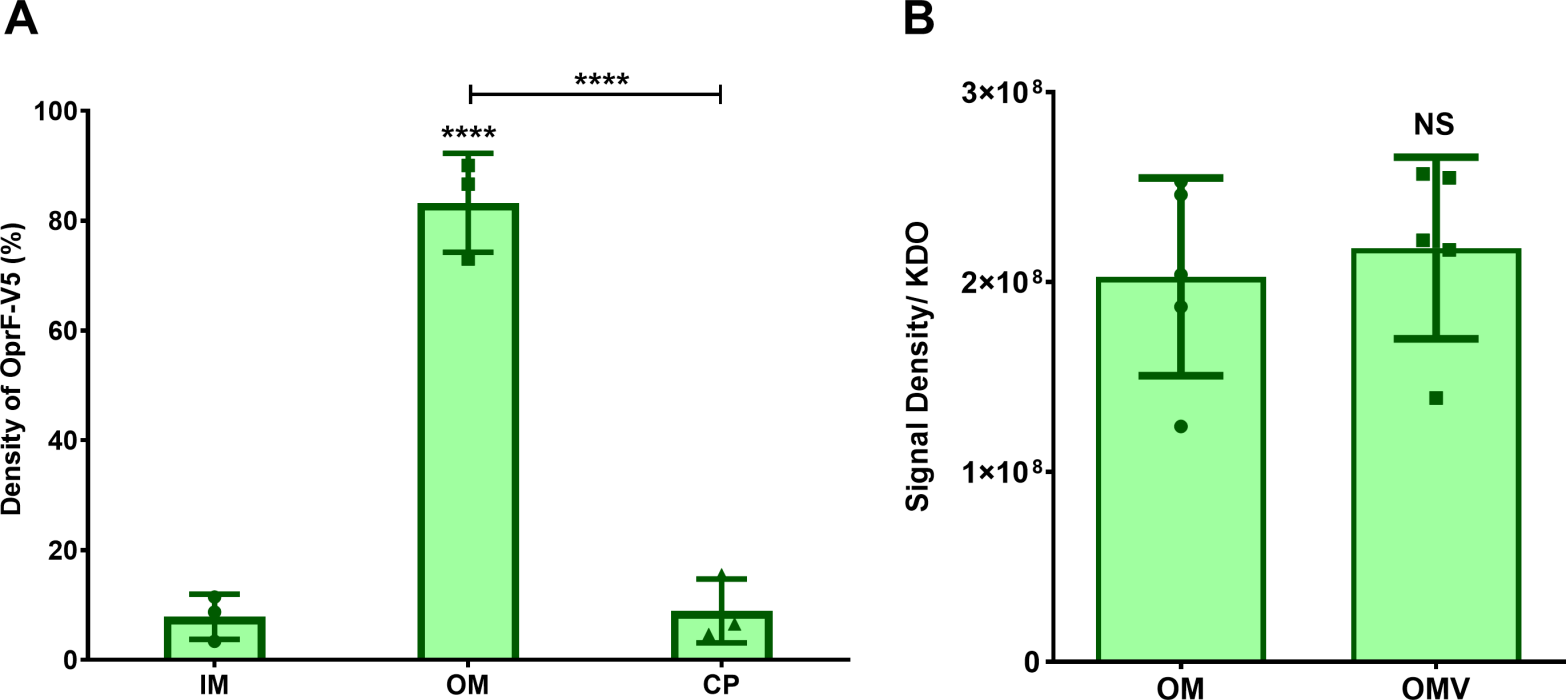
Translocation and sorting of OprF-V5 between the OM and OMVs. (**A**) Proportion of OprF-V5 found in the IM, OM, and CP when expressed in PAO1 ∆*wbpL oprF* V5 (also see Fig. S4). Error bars represent standard deviation. Statistical significance was analyzed by one-way ANOVA, followed by Tukey’s multiple comparisons test (****P < 0.0001; NS, not significant; *n* = 3). (**B**) The amount of OprF-V5 present per unit membrane in OM vs OMVs was assessed by normalizing anti-V5 western blot signal to the concentration of 3-deoxy-D-manno-oct-2-ulosonic acid (KDO) in each fraction (also see Fig. S8). Error bars represent standard deviation. Statistical significance was analyzed by two-tailed unpaired t-test with Welch’s correction (NS, not significant; *n* = 5). Asterisks directly over bars denote differences from WT; asterisks associated with horizontal lines refer to differences between the indicated bars.

To investigate the conformational distribution of OprF in OMVs vs cells, we constructed a variant with a nine amino acid V5 epitope tag inserted at position 312 in the C-terminal region of the protein. This position is known to be protected in the periplasm in the two-domain conformer and exposed on the cell surface in the one-domain conformer (36). Since access to the V5 epitope in the surface-exposed loop of OprF in the one-domain conformer would be important for our analysis, we examined whether the presence of O-antigen on the bacterial surface could interfere with antibody binding. We expressed the OprF-V5 variant in both the O-antigen-lacking PAO1 Δ*wbpL* background and the PAO1 Δ*oprF* background (O-antigen present). It is important to note that the presence or absence of O-antigen is known to have no effect on the level of OMV production in *P. aeruginosa* (48). OMVs were harvested from both strains, and identical samples were analyzed by dot-blot for surface-exposed anti-V5 antibody signal and by western blot for total OprF-V5. Figure 5 clearly shows that the presence of O-antigen reduced signal from the surface-exposed V5 epitope of OprF, despite the presence of equal amounts of OprF-V5 protein. We therefore chose to go forward using the Δ*wbpL* background. As was done for native OprF in the wild-type backgrounds above, we established that OprF-V5 was properly translocated to the OM (Fig. 6A; Fig. S4) and its expression did not affect growth, OMV number, OMV size, or PQS production in the Δ*wbpL* background (Fig. S3C and S5). We then confirmed that the OprF-V5 variant was functional and could complement OMV hypervesiculation in both the PA14 and PAO1 *oprF*-null strains (Fig. S6 and S7).

Before investigating differences in OprF conformation in OMVs vs the OM, it was critical to understand whether OprF is preferentially sorted into or excluded from OMVs. OMVs were purified, and the amount of OprF-V5 was quantified by western blot and normalized to the amount of 3-deoxy-D-manno-oct-2-ulosonic acid (KDO) in the sample. KDO is a sugar unique to the inner core oligosaccharide of LPS, making its quantification a common proxy for the amount of LPS present in a sample (49). Extensive work in the OMV field has also confirmed that OMVs are derived from the OM and are therefore very similar in lipid composition (50–52). This normalization strategy allowed us to assess how much OprF was present “per unit membrane” in a sample. The same procedure was performed on purified OM from sonicated and density-fractionated cells. Figure 6B shows that there is no preferential sorting of OprF between OMVs and the OM. Representative raw western blot data are shown in Fig. S8.

### OprF exists predominantly in the one-domain conformation in OMVs

**Fig 7:**
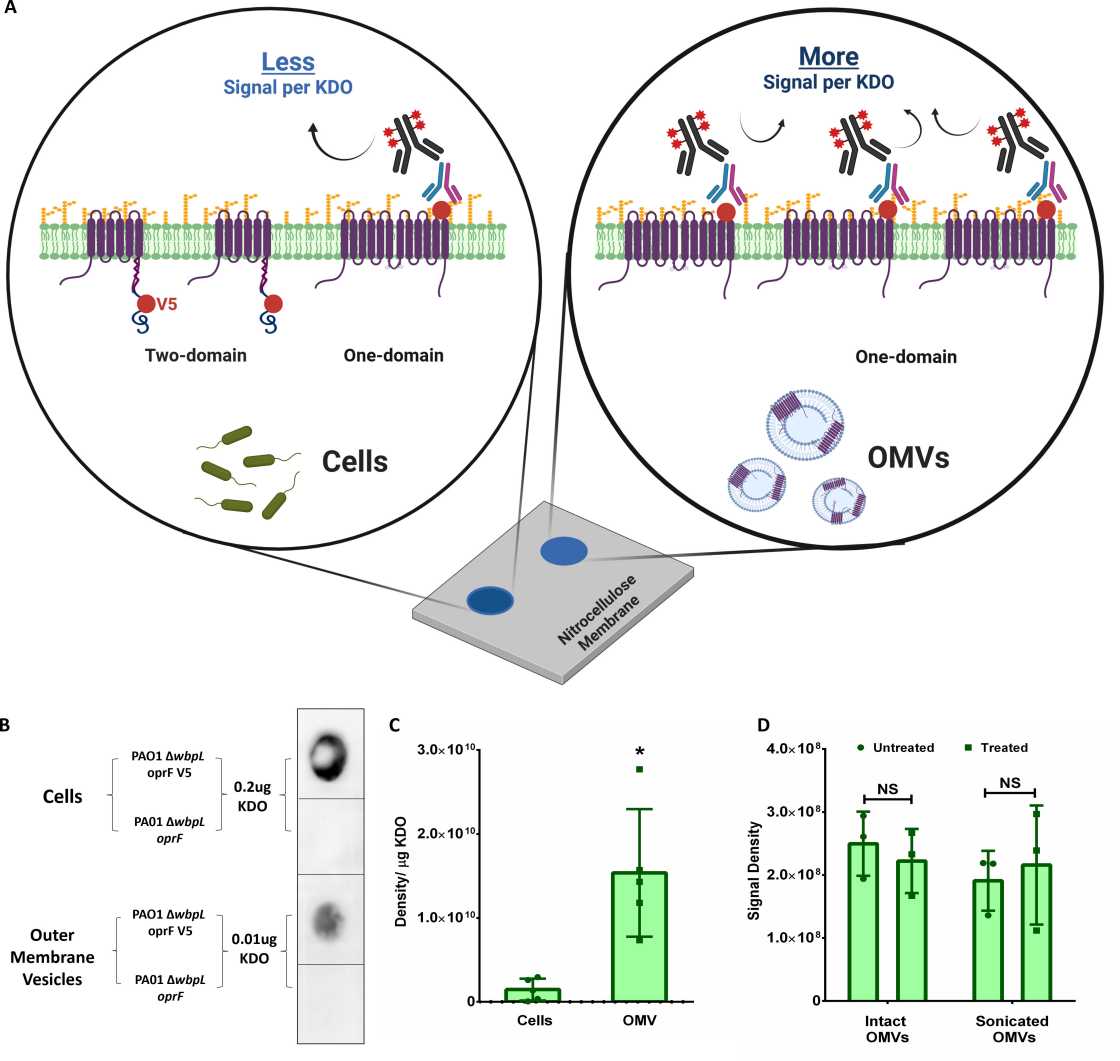
OprF conformation in cells vs OMVs. (**A**) Schematic representation of the dot-blot experiment used to assess OprF conformation in cells vs OMVs. (**B**) Representative dot-blot showing samples of cells and OMVs deposited to reach a comparable anti-V5 signal. The amount of total membrane spotted (0.2 vs 0.01 µg KDO) is depicted (also see Fig. S9). Negative controls expressing untagged OprF are also shown. (**C**) Normalized density of anti-V5 signal per KDO for cells vs OMVs from the dot-blot analysis. The level of accessibility of V5 epitope to exogenous antibody represents the amount of OprF present in the one-domain conformation. Error bars represent standard deviation. Statistical significance was analyzed by two-tailed unpaired *t*-test with Welch’s correction (*P < 0.05; *n* = 5). (**D**) Proteolysis was performed on intact and sonicated OMVs to assess whether the C-terminus of OprF in OMVs is protected by insertion into the membrane (i.e., folded in the one-domain conformation). Loss of anti-V5 signal after proteinase K treatment would represent degradation of the C-terminal domain (also see Fig. S10). Error bars represent standard deviation. Statistical significance was analyzed by multiple *t*-tests which were corrected using the Holm-Sidak method (NS, not significant; *n* = 3).

We continued the use of our dot-blot assay to investigate OprF conformation. Purified OMVs and whole cells were deposited onto nitrocellulose film. The film was then probed using an anti-V5 antibody to quantify the amount of V5 epitope exposed on the surface of OMVs vs cells (Fig. 7A). The amount of membrane, assessed as [KDO], was titrated for each sample so that spots with comparable antibody signal could be compared (Fig. 7B; Fig. S9). The amount of KDO required to achieve equal signal intensity was then measured, allowing us to compare the relative amount of one-domain OprF present per unit membrane in OMVs vs OM. Figure 7C shows that OMVs contain 10.5-fold more one-domain OprF than the cellular OM, while we previously established that the level of total OprF is equivalent in these two fractions. This result strongly suggests that the conformational distribution is flipped from nearly all two-domain OprF in the OM to nearly all one-domain OprF in OMVs.

### Protease protection assay corroborates one-domain conformation of OprF in OMVs

To corroborate our finding that the one-domain conformer predominates in OMVs, we performed a proteolysis protection assay on purified OMVs. The C-terminal region of two-domain OprF is protected in the periplasm (and therefore the OMV lumen) against exogenous protease but becomes susceptible to degradation if protease is allowed access (see Fig. 1 and reference 36). Figure 7D and Fig. S10 show that exogenous protease treatment had no effect on the OprF-V5 signal in intact OMVs, as expected. Importantly, the V5 epitope was also fully protected in the sonicated OMV sample, demonstrating that the C-terminal domain is inserted into the membrane in OMVs. This supports the proposal that the vast majority of OprF in OMVs is present in the one-domain conformation.

## DISCUSSION

Vesicular trafficking is now understood to occur in all domains of life (53, 54). Eukaryotes employ sophisticated systems involving clathrin, caveolin, dynamin, ESCRT proteins, SNAREs, etc. In archaea, ESCRT-related proteins have also been implicated in extracellular vesicle formation (55, 56). Although dynamin-like proteins have recently been identified in some bacterial species (57, 58) and have been associated with secretion of proteins thought to be OMV cargo (59, 60), very little is known about whether proteins contribute specifically to OMV formation. A major question in the field, leading to several competing models (1, 61), has rather been to understand how bacteria could achieve vesicle budding in the absence of eukaryotic-like protein systems. We propose that prior to the evolution of sophisticated protein systems for membrane bending and scission, Gram-negative bacteria developed a program for vesicular transport based upon membrane curvature manipulation (governed by small molecule interactions [23] or lipid sorting [19, 20]) in concert with a mechanism to selectively release patches of the OM from their underlying connections to PG. These “loosened” patches would then be free to bud under the influence of their own continued curvature induction or would be aided by periplasmic turgor (18, 62). With this work, we have uncovered a mechanism by which *P. aeruginosa* can selectively detach certain regions of the OM to be released as OMVs, providing the missing link that could unify many contributing inputs into a generalized model of OMV biogenesis across species.

OM-PG connectivity has long been implicated in OMV formation (16, 25, 26). Equally important, however, must be the *distribution* of OM-PG connections. Tethering of the OM in specific locations must become loose enough for an OMV to form in that spot. OM-PG anchoring proteins are generally excluded from OMVs (27, 50), with the notable exception of OmpA family proteins (63–65). At a minimum, this suggests that OmpA family proteins are present at the sites of OMV formation. Deatherage et al. observed in *Salmonella* that the hypervesiculation phenotype of an *ompA* mutant could not be complemented by overexpression of Braun’s lipoprotein (which provides the most abundant OM-PG linkages in *Salmonella*), hinting at a special role for OmpA in OMV formation (25). Wessel et al*.* (38) carried out a similar analysis in *P. aeruginosa* (where the OmpA homolog is OprF). Deletion of *oprF* resulted in the expected hypervesiculation, but the phenotype was rescued when PQS biosynthesis was simultaneously blocked (38). To us, this solidified the idea that hypervesiculation in *oprF*/*ompA* knockouts is not due to random membrane shedding, but rather is tied to the regulated biosynthesis of OMVs (which in *P. aeruginosa* is initiated by PQS through the bilayer-coupled model [23]). Further conclusions from that work are difficult to draw because of the confounding observation that their *oprF* mutant overproduced PQS (38). Whether due to differences in expression plasmid, strain background, or growth conditions, we did not observe this confounding effect in either our PA14 or PAO1 experiments.

We sought to investigate whether hypervesiculation in *oprF*-null mutants was the consequence of specific loss of OprF-PG connections, rather than general membrane disruption caused by the loss of an abundant OM protein. We first confirmed that hypervesiculating strains did not experience increased cell lysis. We then used a targeted strategy to show that two OprF variants engineered to lack the PG-binding motif (29, 37) were unable to restore WT OMV production in the *oprF*-null mutant background. Vesicle production in the C_del_ variant strain was not different from that of the *oprF* mutant, but interestingly, the PG_del_ variant appeared to partially rescue OMV production. We attribute this to the fact that we deleted only 12 of the 18 amino acids identified as the PG-binding motif (37) because topology studies (66) raised the possibility that full deletion might disrupt an integral membrane region involved in folding into the one-domain conformer. This decision may have resulted in the ability of the PG_del_ variant to retain weak PG binding affinity in the two-domain conformer. Because the native OprF expressed *in trans* was capable of full rescue, we conclude that the *oprF* hypervesiculation phenotype is due to the lost ability of the protein to interact with PG and not because of any indirect effect of deleting an abundant OM protein.

To examine OprF conformation in purified OMVs and live cells, we expressed a variant OprF with a small epitope tag at a position that will only be exposed to the extracellular surface when the protein folds into the one-domain conformation (36, 66). This allowed us to interrogate the amount of OprF in the one-domain conformation in each case using a simple dot-blot. Before we could undertake this straightforward approach, it was necessary to understand whether OprF was sorted preferentially into OMVs and to find a way to normalize the amount of OprF present in OMV vs whole cell samples. By probing for the protein using Western blot and comparing that signal to the amount of LPS (KDO sugar) in each sample, we established that the protein is equally sorted between OMVs and the OM. To our knowledge, this represents the first quantitative analysis of OMV protein sorting based on abundance per unit membrane vs the OM.

With the understanding that the amount of OprF per unit membrane is equivalent in OMVs and cells, we went on to test the proportion of the protein present in the one-domain conformation in each case. The extremely low signal-to-KDO ratio of whole cells confirmed that the vast majority (>90%) (35, 36) of OprF in the OM of cells is in the PG-attached two-domain conformer. The signal-to-KDO ratio for the OMVs, on the other hand, was more than 10-fold increased vs whole cells. We supported this finding using a protease protection assay, which confirmed that nearly all OprF in OMVs was folded in the fully protected one-domain conformer. This demonstrates a dramatic reversal of the conformational distribution of OprF in OMVs and establishes that OM regions released as OMVs in native cells have very low OprF-PG connectivity. Our results are consistent with the recent observations of Orench-Rivera et al*.*, who observed preferential packaging of truncated vs full-length OmpA into *E. coli* OMVs (67).

Our discovery that OprF is found in the “open” one-domain conformation in OMVs has important consequences for OMV physiology. The one-domain conformer folds as a non-specific porin, suggesting that OMVs would be much more permeable than the OM. OMVs have previously been shown to effectively degrade exogenously added antibiotics despite the fact that the antibiotic-degrading cargo enzymes are protected inside the vesicles (68, 69). Increased permeability owing to the dominant one-domain conformer in OMVs explains this. In the biofilm context, OMVs are an abundant component and have been reported to interact with the extracellular matrix (70–72). It has been speculated that an OprF-LecB-Psl tripartite interaction may stabilize OMVs in the matrix and retain them in the biofilm (73). Our finding that OprF exists in a different conformation in OMVs vs cells introduces the possibility that OMV OprF may interact with the matrix in a different manner than cellular OprF. This could have major impacts on the mobility of OMVs in the matrix and their ability to transfer cargo between cells or even out of the biofilm altogether.

Another intriguing question concerns *how* OprF comes to acquire such a different conformational distribution in OMVs vs cells. Seminal work characterizing the two conformations of OprF identified four critical cysteine residues and proposed that each conformer arises from a dedicated folding pathway (35, 36). The interpretation of our results according to that model would be that OprF conformation is static, and the selective packaging of the one-domain conformer into OMVs would arise from the directed arrangement of these conformers in regions destined to become OMVs prior to the initiation of OMV formation. In contrast, van der Heijden *et al.* observed that the OmpA transport pore in *Salmonella* could be rapidly opened or closed in response to oxidative stress (74) through a process that appeared to involve the reordering of disulfide linkages in the protein. This presents the exciting possibility that OprF (and homologs) could dynamically switch their conformation and thereby coordinate localized PG-release from regions of the membrane that otherwise would contain >90% two-domain conformers. This question remains an area of intense study in our laboratory.

With this work, we discovered a complete reversal of conformational distribution of OprF between the OM of cells and OMVs. This selective packaging is made possible by the unique ability of the OprF protein to adopt both a PG-bound and a PG-unbound conformation. This discovery provides a mechanistic explanation for previous reports highlighting the particular importance of proteins like OprF in the vesicle formation process. Integrating this new information into what is already known about the process, we propose that OMV biogenesis proceeds through at least two distinct phases: (i) membrane curvature induction, possibly initiated in different ways under different conditions; and (ii) OM release mediated by the removal of location-specific OprF-PG linkages. We dub this second phase the “Latch Model” of OMV biogenesis. Owing to the broad conservation of the OmpA family (OprF homolog) proteins among bacteria, this model for later steps in OMV formation may serve as a common pathway to advance curvature events that were initiated by small molecule insertion (23), lipid remodeling (19, 20), stress events (17, 18), etc., onward to the final stages of vesicle packaging and release. From a broader evolutionary perspective, this work also helps to shed light on how the ubiquitous process of extracellular vesicle formation can proceed using simple protein systems in the energy-poor environment of the bacterial periplasm.

## MATERIALS AND METHODS

### Bacterial strains and general growth conditions

The bacterial strains and plasmids used are listed in Table S1. *P. aeruginosa* and *E. coli* cultures were routinely cultured in Lysogenic Miller broth/agar (Fisher Scientific). For all other purposes, the cultures were grown in Brain Heart Infusion (BHI) broth (Fisher Scientific). When necessary, media were supplemented with arabinose (Thermo) at 1% (wt/vol), gentamicin (Amresco) at 20 μg/mL for *E. coli* and 50 μg/mL for *P. aeruginosa*, or with carbenicillin (IBI Scientific) at 250 μg/mL for *P. aeruginosa* strains.

### DNA manipulations

Restriction endonucleases and T4 DNA ligase were purchased from New England Biolabs. DNeasy Blood and Tissue Kits (Qiagen) were used to isolate chromosomal DNA from *P. aeruginosa*. EconoSpin spin columns for DNA (Epoch Life Science) were used to isolate plasmid DNA and genomic DNA fragments. DNA was amplified using Platinum *Taq* Polymerase High Fidelity (Invitrogen) or AccuPrime Pfx DNA Polymerase (Thermo Fisher Scientific).

### Construction of *oprF* PG_del_, *oprF* CT_del_, and *oprF* V5 mutants

The *oprF* PG_del_ construct was made by splicing overlap extension PCR (SOE PCR) (75) using PA14 genomic DNA and the SOE primers listed in Table S2. The *oprF* C_del_ construct was created using PA14 genomic DNA as a template with the *oprF* forward and oprF C_del_ reverse primers (Table S2). To create the *oprF* V5 construct, the codon for alanine (5′-GCT-3′) at position 312 in the protein sequence was replaced by the sequence encoding the V5 short tag in the reverse primer (Table S2). PAO1 genomic DNA was used as a template. The final PCR products for *oprF* PG_del_, *oprF* CT_del_, and *oprF* V5 were purified and digested using XbaI and SacI restriction enzymes to allow for cloning into pMJT-1. Final sequences were verified using the Seq primers listed in Table S2. The plasmids were eventually purified from *E. coli* DH5α and electroporated into either the PA14 *oprF*::Tn, PAO1 ∆*oprF*, or PAO1 ∆*wbpL* strains. Transformants were screened using LB agar containing 250 µg/mL carbenicillin.

### Purification of OMVs

Cultures of each strain were inoculated at a starting OD_600_ of 0.01 into 30 mL of fresh BHI medium supplemented with or without 250 µg/mL carbenicillin and 1% (wt/vol) arabinose for induction of the genes expressed through pMJT-1 plasmid. Cultures were incubated at 37°C with shaking (250 rpm) until early stationary phase. Cells were separated from supernatants by centrifugation at 15,000 × *g* for 15 min at 4°C (Thermo Fisher Fiberlite F15s-8x50c rotor). The supernatant was collected, and any remaining cells were removed by filtration through a 0.45 μm polyethersulfone syringe filter. The filtered supernatant was centrifuged at 209,438 × *g* for 90 min at 4°C (Thermo S50-A rotor). The pelleted OMVs were resuspended in 1 mL MV buffer (50 mM Tris, 5 mM NaCl, 1 mM MgSO_4_, pH 7.4).

### OMV concentration and size quantification

OMV number and size distribution were determined using the Malvern NanoSight NS300 instrument along with Nanoparticle Tracking and Analysis software (NTA 3.2) (22). To obtain 20 to 100 particles per frame (as per the manufacturer’s instructions), OMV samples were diluted 1:400 to 1:3,200. The camera level was manually set to 12 with a gain of 1. Using different fields of view, each sample was analyzed three times for 30 s at 25°C. OMV concentration was normalized to the OD_600_ of the extracted culture.

### PQS quantification

PQS was quantified as previously described (13). Briefly, PQS was extracted from bacterial cultures in 1:1 acidified ethyl acetate (0.1 mL/L acetic acid) before the organic phase (containing PQS) was removed and dried under nitrogen gas. Dried samples were resuspended in 25 µL optima grade methanol (Fisher) and spotted onto a straight-phase phosphate-impregnated TLC plate (EM Biosciences). PQS standards (Sigma) (100–500 µM) were spotted on the same plate. The TLC was developed using 95:5 dichloromethane-methanol as the mobile phase. PQS has intrinsic fluorescence, which was excited under long-wave UV light and visualized (UVP Gel Doc-It2). Digital images were captured and analyzed using VisionWorks LS Image Acquisition and Analysis software.

### Cell fractionation

This protocol was adapted from our previously published work (46). All the test strains were grown in 500 mL BHI for 18 h at 37°C and 250 rpm. Cells were harvested by centrifugation at 15,000 *× g* for 15 min at 4°C (Thermo Fisher Fiberlite F15s-8x50c rotor). The cell pellets were washed in 100 mL of 30 mM Tris-HCl buffer (pH 8.0) and centrifuged again at 10,000 *× g* for 10 min at 4°C. Washed pellets were then resuspended in 30 mL of 20% sucrose (prepared in 30 mM Tris-HCl buffer, pH 8.0). The cells were lysed via sonication in the presence of 10 µL/mL HALT protease inhibitor cocktail plus EDTA (Thermo Fisher). Unlysed cells were removed by centrifugation at 10,000 *× g* for 10 min. The supernatant was filtered through a 0.45 µm syringe filter, and the total membrane fraction was collected through ultracentrifugation at 100,000 *× g* for 1 h. The pellet obtained was resuspended in 1 mL of 20% sucrose (prepared in 30 mM Tris-HCl buffer, pH 8.0). The supernatant was saved to be used as a cytoplasmic fraction for downstream processes. To separate inner and outer membranes further, the pellet in 20% sucrose was layered on top of a sucrose gradient composed of 3 mL of 70% (wt/vol) sucrose under 8 mL of 60% (wt/vol) sucrose. The gradient was centrifuged at 32,700 rpm for 18 h at 4°C (Beckman SW41-Ti rotor). After the centrifugation, 500 µL fractions were collected from the top of the gradient. Sucrose concentration was reduced by diluting fractions into 30 mM Tris-HCl buffer, pH 8.0, and samples were centrifuged at 100,000 *× g* for 1 h (Thermo S120AT2 rotor). The washed pellets were resuspended and stored in 500 µL of 30 mM Tris-HCl buffer, pH 8.0.

### 3-Deoxy-D-manno-2-octulosonic acid assay

This protocol was modified from previous methods (49). Twenty-five microliters of sample and KDO standards were mixed 1:1 with 0.5 M H_2_SO_4_ and boiled for 15 min. After cooling at room temperature for 10 min, 25 µL of 0.1 M periodic acid was added to each sample. Samples were vortexed and incubated at 25°C for 10 min, after which 100 µL of 0.2 M sodium arsenate in 0.5 M HCl was added. The samples were vortexed, and 400 µL of freshly prepared 0.6% thiobarbituric acid was added. Each sample was boiled for 10 min and then cooled at room temperature for 30–40 min. After the addition of 750 µL of acidified n-butanol, the organic layer was recovered, and absorbance was measured at 552 and 509 nm. The measurements at 509 nm were subtracted from the measurements at 552 nm, and the resulting values were compared to identically treated KDO standards.

### Succinate dehydrogenase assay

A reaction mixture of 200 µL (50 mM Tris-HCl [pH 8.0], 4 mM potassium cyanide, 20 mM disodium succinate) was preincubated for 5 min at room temperature in the wells of a 96-well plate. After this, 10 µL of sample was added to the mixture and further incubated for 5 min at room temperature. To initiate the reaction, 2,6-dichlorophenolindophenol and 0.2 mM phenazine methosulfate (PMS) were added to the mixture, and the SDH activity was measured by recording the absorbance at 600 nm over time at 25°C (Tecan Infinite M-200 Pro).

### Immunoblotting analysis

Total protein (0.5–1 µg), based on Bradford assay of the IM, OM, and CP fractions isolated from bacteria grown in BHI, was separated by SDS-PAGE (12% gel). Proteins were transferred to a PVDF membrane (Bio-Rad Trans-Blot Turbo) and blocked in 5% skim milk in Tris-buffered saline with 0.1% Tween-20. The membrane was then incubated with anti-OprF MA7-1 antibody (1:10000; kind gift of Dr. R. E. W. Hancock) (66). After washing, the membrane was incubated with HRP-conjugated donkey anti-mouse IgG antibody (1:1,000) (Jackson Immuno Research), washed again, and exposed to Super Signal West Pico Chemiluminescent substrate (1:1,000) (Thermo). Bands were visualized using the Gel Doc-It2 system (UVP) and quantified using VisionWorks LS software.

### Protease protection assay

To determine the conformational state of the modified OprF PG_del_ protein, proteolysis of membrane-associated OprF was performed using a modification of the protocol of De Mot and Vanderleyden (76). Outer membrane fractions from PA14 *oprF*::Tn *oprF* PG_del_ and PAO1 ∆*oprF oprF* PG_del_ strains, prepared as described above, were suspended in 500 µL of 30 mM Tris-HCl, pH 8.0. Half of each sample was incubated with 100 µg/mL proteinase K (Epoch Life Science) at pH 8.0 at 37°C for 30 min. The reaction was stopped by adding PMSF at a final concentration of 0.3 mg/mL. The other half of each sample was used as a control to compare with the protease-treated samples. Total protein was quantified in the treated and untreated samples using the Bradford assay. Proteolysis results were analyzed by western blot as described above.

To determine the conformational state of OprF V5 in the vesicles, OMVs from PAO1 ∆*wbpL oprF* V5 were harvested as described above and split into four identical sub-fractions of 100 µL. The first two reaction mixtures served as “intact” OMV samples. One of these OMV samples was treated with 100 µg/mL proteinase K, while the other one functioned as an untreated control. The third and fourth reaction mixtures served as “sonicated” OMV samples. One of these OMV samples was incubated with proteinase K enzyme while being sonicated in order to expose the lumen of the vesicles to the enzyme. The other sample was sonicated in the absence of proteinase K. Proteinase K treatment was conducted at 37°C for 30 min, after which the reaction was stopped by adding trichloroacetic acid to a final concentration of 10% (wt/vol). Samples were diluted into 1 mL 30 mM Tris buffer (pH 8.0) and ultracentrifuged at 100,00 × *g* for 1 h (S120-AT2 fixed angle rotor). The pellet was resuspended in 100 µL of 30 mM Tris buffer (pH 8.0), and KDO assays were performed (as above) to analyze the total amount of outer membrane present in each sample. The samples were loaded based on equal amounts of KDO (0.2 µg/lane), separated by SDS-PAGE (12%), and analyzed by western blot as described above (but with anti-V5 primary antibody).

### OprF sorting between OM and OMVs

PAO1 ∆*wbpL oprF* V5 was grown at 37°C (250 rpm) in 500 mL BHI, supplemented with 1% Arabinose and 250 µg/mL carbenicillin, until early stationary phase. Cells were collected by centrifugation at 15,000 × *g* for 15 min, and the supernatant was used to harvest vesicles (described above) after being concentrated to ≤50 mL by tangential flow filtration. Cells were lysed by sonication, and membranes were fractionated as described above. The OM and OMV samples obtained were loaded based on equal amounts of KDO (0.2 µg/lane) and separated by SDS-PAGE (12%). The amount of OprF-V5 present in each fraction (normalized to KDO) was analyzed by western blot using an anti-V5 antibody as described above.

### Dot-blot assay

To assess whether the presence of O-antigen hinders binding of anti-V5 antibody, OMVs were first harvested from four strains (PAO1 ∆*wbpl* pMJT1-*oprF*, ∆*wbpl* pMJT1-*oprF V5*, ∆*oprF* pMJT1-*oprF*, and ∆*oprF* pMJT1-*oprF V5)*. Each of the OMV samples (10 µL) normalized to contain 0.01 or 0.05 µg KDO was spotted on a pre-wetted (in Tris-buffered saline with 0.1% Tween-20) nitrocellulose membrane and dried for 1 h at RT. Dot-blot analysis was performed using anti-V5 antibody (1:500) (Thermo) and HRP-conjugated donkey anti-mouse IgG antibody (1:5,000) (Jackson Immuno Research), followed by exposure to Super Signal West Pico Chemiluminescent substrate (1:1,000) (Thermo). Bands were imaged using the ChemiDoc Imaging System (Bio-Rad). The experiment was performed in triplicate. Western blots were also performed on the OMV samples as described above (immunoblotting analysis), ensuring that the total amount of OprF V5 present in each sample was the same for the dot-blots and western blots.

Similarly, to assess the conformational state of OprF-V5 in bacterial cells vs OMVs, cells were collected (described above) from 1 mL of PAO1 ∆*wbpL oprF* V5 culture, grown in 500 mL BHI supplemented with 1% arabinose and 250 µg/mL carbenicillin until early stationary phase at 37°C (250 rpm). The cells were stored in the presence of HALT protease inhibitor cocktail plus EDTA (Thermo) at 4°C for downstream processing. The rest of the bacterial culture was used to harvest OMVs (described above). PAO1 ∆*wbpL oprF* bacterial cells and vesicles were harvested identically to be used as negative controls (no V5 epitope). KDO assay (described above) was used to determine the amount of outer membrane present in both cell and OMV samples. Bacterial cells and OMV samples (10 µL) with a total of 0.2 and 0.01 µg KDO, respectively, were then spotted on a pre-wetted (in Tris-buffered saline with 0.1% Tween-20) nitrocellulose membrane and dried for 1 h at RT. The dot-blot was visualized using antibodies and chemiluminescence analysis as described above. The antibody signal obtained from PAO1 ∆*wbpL oprF* bacterial cells and vesicles (negative controls) was subtracted from the signal obtained from PAO1 ∆*wbpL oprF* V5. The final signal density from bacterial cells and OMV samples was then normalized to the amount of KDO present in each sample.

## SUPPLEMENTAL MATERIAL

Supplemental Material mbio.01017-26-s0001.docx Figures S1 to S10; Tables S1 and S2.

ASM does not own the copyrights to Supplemental Material that may be linked to, or accessed through, an article. The authors have granted ASM a non-exclusive, world-wide license to publish the Supplemental Material files. Please contact the corresponding author directly for reuse.
