# Supplementary material for: OprF functions as a latch to direct outer membrane vesicle release in Pseudomonas aeruginosa
Source: mBio. 2026 Jul 16;17(8):e01017-26. doi: 10.1128/mbio.01017-26 (PMC13463901; doi:10.1128/mbio.01017-26)
Supplement: Supplementary file 1 [file mbio.01017-26-s0001.docx]

**Supplemental Information for**

OprF functions as a latch to direct Outer Membrane Vesicle release in Pseudomonas aeruginosa.

Shrestha Mathur^†^, Citrupa Gopal^†^, Susan K. Erickson, Leah R. Goldberg, Sonia Hills, Abigail G.B. Radin, and Jeffrey W. Schertzer*

Department of Biological Sciences, Binghamton University, Binghamton, NY 13902

Binghamton Biofilm Research Center, Binghamton University, Binghamton, NY 13902

Department of Cell Biology and Molecular Genetics, Maryland Pathogen Research Institute, University of Maryland, College Park, MD, 20742

^†^ Authors contributed equally to this work

*Corresponding Author

**Corresponding Author Email:** [jschertz@binghamton.edu](mailto:jschertz@binghamton.edu)

**Supplemental Information includes:**

Figures S1 to S10

Tables S1 and S2

##### Fig. S1. Confirmation of separation of sub-cellular fractions. KDO and SDH assay on inner membrane (IM), outer membrane (OM), and cytoplasmic (CP) fractions of (A) PA14 oprF complement, (B) PA14 oprF PG_del_ variant, (C) PA14 oprF C_del_ variant (D) PAO1 oprF complement, (E) PAO1 oprF PG_del_ variant, and (F) PAO1 oprF C_del_ variant. Error bars represent standard deviation. Statistical significance was analyzed by Two-way ANOVA followed by Sidak's multiple comparisons test between IM, OM, and CP (****p < 0.0001) and n = 3.

**Fig. S2. Western Blot of OprF in different fractions.** Western blot showing OprF detected by antibody specific for the N-terminal region of the protein in outer membrane (OM), inner membrane (IM) and cytoplasmic (CP) fractions collected from (A) PA14 *oprF::Tn* complemented by different variant proteins and (B) PAO1 Δ*oprF* complemented by different protein variants. Black arrows mark full length protein for each construct. The figures are representative of three independent experiments.

**Fig. S3. Growth of bacterial strains**. The optical density measured over time for all the strains in the (A) PA14, (B) PAO1 and (C) PAO1 ∆*wbpL* backgrounds. Panels (D) and (E) show the growth of strains complemented with oprF-V5. Data are plotted as mean ± standard deviation (n = 3). Cultures were grown in BHI with shaking (250rpm) at 37°C.

Fig. S4. Confirmation of membrane separation and presence of OprF-V5 in outer membrane. Separation of outer membrane (OM), inner membrane (IM) and cytoplasmic fractions (CP) was confirmed for (A) PAO1 ∆*wbpL* *oprF* and (B) PAO1 ∆*wbpL* *oprF* V5 by measuring the presence of KDO sugar and SDH enzyme activity. Error bars represent standard deviation. Statistical significance was analyzed by Two-way ANNOVA followed by Sidak's multiple comparisons test between IM, OM, and CP (****p < 0.0001, NS= not significant) and n = 3. (C) Western blot using anti-V5 antibody on inner membrane (IM), outer membrane (OM) and cytoplasmic (CP) fraction from PAO1 ∆*wbpL* *oprF* and PAO1 ∆*wbpL* *oprF* V5. The figure is a representative of three independent experiments. Asterisks directly over bars denote differences from IM; asterisks associated with horizontal lines refer to differences between the indicated bars.

Fig. S5. Analysis of PAO1 ∆*wbpL* strains. OMVs from each strain were harvested at early stationary phase and analyzed for vesicle number (A) and vesicle size (B) by nanoparticle tracking analysis. Error bars represent standard deviation. Statistical significance was analyzed by One-way ANOVA followed by Dunnett’s multiple comparisons test and n = 3 (NS = not significant). (C) PQS was extracted and quantified from each culture using thin layer chromatography and densitometry analysis. Error bars represent standard deviation. Statistical significance was analyzed by One-way ANOVA followed by Dunnett’s multiple comparisons test and n = 3 (NS = not significant). (D) Succinate dehydrogenase enzyme activity was measured over time in the OMVs from all the strains and compared to lysed bacterial cells (positive control). The figure is a representative of three independent experiments.

Fig. S6. Analysis of PA14 *oprF*::Tn strains complemented using *oprF*-V5. OMVs from each strain were harvested at early stationary phase and analyzed for vesicle number (A) and vesicle size (B) by nanoparticle tracking analysis. Error bars represent standard deviation. Statistical significance was analyzed by One-way ANOVA followed by Dunnett’s multiple comparisons test and n = 3 (NS = not significant). (C) PQS was extracted and quantified from each culture using thin layer chromatography and densitometry analysis. Error bars represent standard deviation. Statistical significance was analyzed by One-way ANOVA followed by Dunnett’s multiple comparisons test and n = 3 (NS = not significant). (D) Succinate dehydrogenase enzyme activity was measured over time in the OMVs from all the strains and compared to lysed bacterial cells (positive control). The figure is a representative of three independent experiments.

Fig. S7. Analysis of PAO1 Δ*oprF* strains complemented using *oprF*-V5. OMVs from each strain were harvested at early stationary phase and analyzed for vesicle number (A) and vesicle size (B) by nanoparticle tracking analysis. Error bars represent standard deviation. Statistical significance was analyzed by One-way ANOVA followed by Dunnett’s multiple comparisons test and n = 3 (NS = not significant). (C) PQS was extracted and quantified from each culture using thin layer chromatography and densitometry analysis. Error bars represent standard deviation. Statistical significance was analyzed by One-way ANOVA followed by Dunnett’s multiple comparisons test and n = 3 (NS = not significant). (D) Succinate dehydrogenase enzyme activity was measured over time in the OMVs from all the strains and compared to lysed bacterial cells (positive control). The figure is a representative of three independent experiments.

**Fig. S8. Sorting of OprF between OM and OMVs.** Outer membrane (OM) fractions and vesicles harvested from PAO1 ∆*wbpL* V5 were loaded and separated on a 12% SDS-PAGE gel based on equal amount of KDO (0.2 µg/lane). Samples were transferred to PVDF membrane before being probed using anti-V5 antibody. The figure is representative of five independent experiments.

**Fig. S9. Raw OprF-V5 signal in cells vs. OMVs.** Densitometry analysis after incubation with anti-V5 antibody of dot-blots shown in Fig 7B showing comparable signal density between cells and OMVs spotted based on total KDO of 0.2 µg and 0.01 µg per spot, respectively. Error bars represent standard deviation. Statistical significance was analyzed by Two- tailed unpaired t-test with Welch’s correction. (NS=not significant) and n=3.

**Fig. S10.** **Protection of OprF C-terminal domain in OMVs.** Western blot using anti-V5 antibody on intact and sonicated OMVs which were either not treated or treated with proteinase K enzyme (quantified in Fig 7D). All samples were loaded based on equal amount of outer membrane (KDO per lane = 0.02 µg). The image is representative of three independent experiments.

**Table S1. Strains and Plasmids used in this study.**

| Strain or Plasmid | Description | Reference |
| --- | --- | --- |
| **Strain** | | |
| ***Escherichia coli*** | | |
| DH5α | F– Φ80lacZΔM15 Δ(lacZYA-argF) U169 recA1 endA1 hsdR17 (rK–, mK+) phoA supE44 λ– thi-1 gyrA96 relA1 | (1) |
| ***Pseudomonas aeruginosa*** | | |
| PA14 | Wild type *Pseudomonas aeruginosa* strain | (2) |
| PA14 *oprF*::*Tn* | PA14 *oprF::Mar2xT7* (Gm^r^) | (2); kind gift from Dr. Karin Sauer |
| *P. aeruginosa* MPA01 | Wild type *Pseudomonas aeruginosa* strain | (3) (4); kind gift from Dr. Karin Sauer |
| PAO1 ∆*oprF* | *oprF* clean deletion in MPAO1 background | kind gift from Dr. Boo Shan Tseng |
| PA01 ∆*wbpL* | Wild type *Pseudomonas aeruginosa* strain | (5), (6); kind gift from Dr. J.S. Lam |
| **Plasmids** | | |
| Strain or Plasmid | Description | Reference |
| pMJT-1 | Carb^R^; *araC-pBAD* expression vector | (7) |
| pMJT-1 *oprF* | Carb^R^; pMJT-1 derived *oprF* over expression vector | This study |
| pMJT-1 *oprF* PG_del_ | Carb^R^; pMJT-1 derived *oprF N_265_-V_276_ del* over expression vector | This study |
| pMJT-1 *oprF* CT_del_ | Carb^R^; pMJT-1 derived *oprF* K_164_-K_326_ *del* over expression vector | This study |
| pMJT-1 *oprF* V5 | Carb^R^; pMJT-1 derived *oprF A_312_IPNPLLGLD* over expression vector | This study |

**Table S2. List of Primers used in this study.**

| Oligonucleotide name | Sequence (5’–3’) *^a^* |
| --- | --- |
| oprF Forward | CGCGCGC*TCTAGA***GACCATCAAGATGGGGATTTAACGGATG** |
| oprF Reverse | GGGGGGGG*GAGCTC***CGATTACTTGGCTTCAGCTTC** |
| oprF PG_del_ SOE 5’ | TCGTTGACCAGTACGTCACG**GTAAGCGTCGGTGCCGACG** |
| oprF PG_del_ SOE 3’ | CGTCGGCACCGACGCTTAC**CGTGACGTACTGGTCAACGA** |
| *oprF* CT_del_ Reverse | GTCAGTCA*GAGCTC*TTA**CGAACCACCGAAGTTGAAGCCGA** |
| *oprF* V5 Reverse | GCAGTCA*GAGCTC***TTACTTGGCTTCAGCTTCTACTTCGGCTTCAACGCGACGGTTGAT**ATCCAGGCCCAGCAGCGGGTTCGGGAT**GCGGCCTTCAGCGG** |
|  |  |
| pMJT-1 Seq Forward | **GACCGCGAATGGTGAG** |
| pMJT-1 Seq Reverse | **GAGCTGATACCGCTCG** |

*^a^* Italics represent regions of enzyme restriction sites. Nucleotides in bold are regions on oligos which allow them to act as PCR primers on appropriate template.

**References**

1.	S. G. N. Grant, J. Jesseet, F. R. Bloomt, D. Hanahan, Differential plasmid rescue from transgenic mouse DNAs into Escherichia coli methylation-restriction mutants (bacterial restriction/DNA methylation/cloning mammalian DNA/heterogeneous transgene expression/insulin gene regulation). *Proc. Natl. Acad. Sci. USA* 87, 4645–4649 (1990).

2.	N. T. Liberati, *et al.*, An ordered, nonredundant library of Pseudomonas aeruginosa strain PA14 transposon insertion mutants. *Proc. Natl. Acad. Sci. U. S. A.* 103, 2833–8 (2006).

3.	M. A. Jacobs, *et al.*, Comprehensive transposon mutant library of Pseudomonas aeruginosa. *Proc. Natl. Acad. Sci. U. S. A.* 100, 14339–44 (2003).

4.	J. Klockgether, *et al.*, Genome Diversity of Pseudomonas aeruginosa PAO1 Laboratory Strains. *J. Bacteriol.* 192, 113–1121 (2009).

5.	H. L. Rocchetta, L. L. Burrows, J. C. Pacan, J. S. Lam, Three rhamnosyltransferases responsible for assembly of the A-band D- rhamnan polysaccharide in Pseudomonas aeruginosa: A fourth transferase, WbpL, is required for the initiation of both A-band and B-band lipopolysaccharide synthesis. *Mol. Microbiol.* 28, 1103–1119 (1998).

6.	K. Murphy, *et al.*, Influence of O Polysaccharides on Biofilm Development and Outer Membrane Vesicle Biogenesis in Pseudomonas aeruginosa PAO1. *J Bacteriol* 196, 1306–1317 (2014).

7.	Y. Kaneko, M. Thoendel, O. Olakanmi, B. E. Britigan, P. K. Singh, The transition metal gallium disrupts Pseudomonas aeruginosa iron metabolism and has antimicrobial and antibiofilm activity. *Journal of Clinical Investigation* 117, 877–888 (2007).
